# CamKII inhibitors reduce mitotic instability, connexon anomalies and progression of the *in vivo* behavioral phenotype in transgenic animals expressing a mutated Gjb1 gene

**DOI:** 10.3389/fnins.2014.00151

**Published:** 2014-06-13

**Authors:** Saleh Mones, Benoit Bordignon, Franck Peiretti, Jean F. Landrier, Burkhardt Gess, Jean J. Bourguignon, Frédéric Bihel, Michel Fontés

**Affiliations:** ^1^Therapy of Genetic Disorders, Faculté de Médecine, EA 4263, Aix-Marseille UniversitéMarseille, France; ^2^INSERM, UMR1062, Nutrition, Obesity and Risk of Thrombosis, Faculté de MédecineMarseille, France; ^3^INRA, UMR1260, Faculté de MédecineMarseille, France; ^4^Department of Sleep Medicine and Neuromuscular Disorders, University Hospital MünsterMünster, Germany; ^5^Laboratoire d'Innovation Thérapeutique, Faculté de Pharmacie, CNRS, UMR7200, Université de StrasbourgIllkirch Graffenstaden, France

**Keywords:** calmodulin kinase, myelination

## Abstract

Mutation in the Gjb1 gene, coding for a connexin (Cx32), is associated with an inherited peripheral neuropathic disorder (X-linked Charcot-Marie-Tooth, CMTX). Our previous work reported that transgenic animals expressing a human Gjb1 transgene present polyploidy and abnormal over-duplication of the centrosome, suggesting a role for Gjb1 in mitotic stability. In this article, we propose mechanisms by which mutations in Gjb1 induce mitotic instability and discuss its potential relation with the CMTX phenotype. We showed that transgenic cells exhibit CamKII over-stimulation, a phenomenon that has been linked to mitotic instability (polyploidy, nuclear volume and centrosome over-duplication), that is reversed by CamKII inhibitors. We also demonstrate that connexon activity is partially restored in transgenic cells with CamKII inhibitors. Our model supports the role for Pim1, a kinase that has been associated with genomic instability in cancers, in genomic instability in Cx32 mutations. Regarding *in vivo* phenotype, we showed that degradation on the rotarod test in our transgenic mice is significantly lowered by treatment with a CamKII inhibitor (KN93). This effect was seen in two lines with different point mutations in GJB1, and stopping the treatment led to degradation of the phenotype.

## Introduction

X-linked Charcot-Marie-Tooth disease (CMTX) is an inherited X-linked peripheral neuropathy (Dyck and Lambert, 1968; Hahn et al., 1990; Martyn and Hughes, 1997), presenting both demyelinating and axonal anomalies (Bergoffen et al., 1993; Hanemann et al., 2003). CMTX is caused by mutations in the gene GJB1 (Bergoffen et al., 1993), located on the proximal long arm of the X chromosome, that encodes connexin 32 (Cx32), a myelin membrane protein found in the PNS and CNS (Scherer et al., 1995). Cx32 is located in gap junctions and forms hexameric hemichannels called connexons (Mese et al., 2007). The docking of two connexons across an intercellular gap triggers the formation of a channel that connects the cytoplasm of adjacent cells (Abrams et al., 2001) allowing exchange of ions, small molecules (<1000 Da) and signaling effectors (Bennett et al., 1991).

Less than 10% of the mutations observed in patients are null alleles (De Jonghe et al., 1999; Abrams et al., 2001; Boerkel et al., 2002; Wang et al., 2004; Bicego et al., 2006). The majority of mutations correspond to a loss of function and could be classified into two categories: mutations affecting cell trafficking, where the Cx32 protein is observed in the endoplasmic reticulum and/or the Golgi but not in the cell membrane (Deschênes et al., 1997; VanSlyke et al., 2000; Yum et al., 2002); and mutations affecting connexon functions, where Cx32 is present in the cell membrane but the connexon remains closed, thus preventing any exchange between adjacent cells (Omori et al., 1996; Yoshimura et al., 1996; Oh et al., 1997).

Models of CMTX have been already described, but either they are based on gene invalidation (Nelles et al., 1996)—a situation rarely observed in humans—or on a molecular construction that puts Gjb1 expression under the control of an exogenous promoter (Sargiannidou et al., 2009). We thus generated transgenic mouse lines with BACs containing human GJB1 genes expressing either the mutation G12S, observed in non-related CMTX families and affecting trafficking of Cx32 (G12S) (Yum et al., 2002) or the mutation S26L, affecting connexon activity (S26L) (Yoshimura et al., 1996; Oh et al., 1997). Five transgenic lines were generated and studied, which led to the observation of genomic instability in transgenic cells and abnormalities of centrosome duplication. These data suggested that Cx32 is involved in the control of mitotic stability. In addition, we noticed that the transgenic animals presented abnormal behavior on the rotarod (Mones et al., 2012).

Calcium ionophores and CamKII over-expression affect mitotic stability leading to centrosome over-duplication (Matsumoto and Maller, 2002) in *Xenopus* oocytes. We hypothesized that CamKII could be a downstream effector of Gjb1 anomalies. To test this, we used two transgenic lines: G2 (two copies of the mutated BAC, presenting a mutation affecting cell trafficking) and S3 (three copies of the mutated BAC, harboring a mutation affecting connexon activity). We observed CamKII over-stimulation in the transgenic cells leading to mitotic instability, and reversal of this instability upon adding CamKII inhibitors. We also observed partial restoration of connexon activity upon addition of CamKII inhibitors in the transgenic lines and in sciatic nerve organ cultures. Furthermore, we demonstrated that degradation of the behavior phenotype of our transgenic mice, observed on the rotarod, is significantly improved by treatment with a CamKII inhibitor (KN93). We extend our hypothesis that Pim1, a kinase that has been associated with genomic instability in cancers (Roh et al., 2003, 2005), could also be involved in genomic instability in Cx32 mutations. In this report, we draw upon the results of our work to discuss the mechanism of Gjb1 mutations in oligodendrocyte maturation and myelination that explains the observations reported in a recent paper relating cellular phenotype and anomalies in glial cells (Waggener et al., 2013).

## Materials and methods

### Ethics statement

Animal experimentation was carried out in CEPA (Advanced Physiological Studies Centre). CEPA has been agreed by French National authorities, that is Departmental Directorate of Protecting People (DDPP) and specifically Veterinary Service (agreement N°A13-055-27). CEPA is in charge of observance of ethical rules of our ethic committee and, in this case, it is not necessary to have a special permit to achieve rotarod performance tests on mice. Animals have been sacrificed using an excess of anesthetic administration (Isoflurane) as recommended by European and National Guidelines in order to minimize suffering.

### Generation of transgenic lines

BAC modifications were generated by Gene Bridges GmbH Heidelberg using recombineering technology. BAC DNA was isolated from preparative pulsed field gels using a modification of a previously described method (Huxley et al., 1996). Transgenic mice were generated using the standard technique of pronuclear injection using C57BL/6J—CBA/Ca F1 mice as donors. Subsequent crosses were to the same F1 mice.

### Cell culture

For the isolation of fibroblasts a small fragment of mouse ear was removed, dipped in alcohol solution, cut into small pieces in a sterile Petri dish in the presence of PBS containing fungicide (fonigizon) diluted 1/250, and transferred to 2-ml tubes containing 1 ml dissociation buffer (DMEM plus 20% FBS, 1 mg/ml BSA, 0.5 mg/ml collagenase, 0.25 mg/ml trypsin and penicillin/streptomycin). The tubes were incubated in a water bath with agitation for 1 h at 37°C. Fibroblast medium (DMEM, 10% FBS, 2 mM Gln, 100 U/ml penicillin, 100 μg/ml streptomycin) was added to each tube and the samples were centrifuged at 400 g for 10 min. The cells were re-suspended in fibroblast medium, seeded into Petri dishes and placed in an incubator at 37°C, 5% CO_2_. The culture medium was replaced every 2 days. When the cultures reached sub-confluence, the cells were trypsinized and expanded into tissue culture flasks. All experiments were performed using cells between passage numbers four and eight.

### Western blotting

Cells were lysed in RIPA buffer (50 mM Tris-Cl pH 7.4, 1% NP40, 0.25% sodium deoxycholate, 0.1% SDS, 150 mM sodium chloride) supplemented with protease and phosphatase inhibitors. The same amounts of protein from each sample were resolved under denaturing and reducing conditions on 4–12% NuPAGE gels (Invitrogen) and transferred to polyvinylidene fluoride membranes. Immunoreactive proteins were revealed by enhanced chemiluminescence with ECL (Perkin-Elmer). An antibody against phosphorylated CamKII (Cell Signaling, catalog number: 3361) was used.

### Nuclear volume evaluation

Nuclei were stained with DAPI. Surface of nuclei stained with DAPI, were evaluated using *ImageJ* software. Average nuclear volume was evaluated using the same software. 100 DAPI stained nuclei were analyzed in 4 independent preparations.

### Centrosome labeling

Cells were grown on glass coverslips for 24 h to allow cultures to reach 80% confluence. To measure the number of centrosomes, cells were fixed with 4% PFA, permeabilized with methanol at −20°C for 8 min and blocked with 0.5% Triton X-100 in PBS for 30 min at RT. To detect γ-tubulin, cells were incubated overnight at 4°C with a mouse anti-γ-tubulin antibody (GTU-88; Sigma) diluted 1/1000 in PBS containing 0.1% milk and 0.05% Triton X-100. After washing, the cells were incubated for 1 h at RT with Cy3-conjugated goat anti-mouse IgG secondary antibody (Caltag Laboratories) diluted 1/2000 in PBS containing 0.1% milk and 0.05% Triton X-100. The preparations were counterstained with DAPI in Vectashield mounting medium (Vector Laboratories). Fluorescent images were acquired with a microscope (Leica DMR) equipped with a PL APO objective.

### Connexon activity

One hundred thousand cells were cultured as described above for 1 day with or without CamKII inhibitors (KN62 or KN93 at a final concentration of 10 μM). Lucifer yellow (LY) was added to the medium (final concentration: 110 μM) and incubated for 2 h. Fluorescence was recorded using a Perkin Elmer Victor 4 microplaque reader (excitation: 405 nM, emission: 535 nM).

### Connexon activity in nerves

This methods has been derived to a recently methods developed in the laboratory (Bordignon et al., 2013). Sciatic nerves of WT or S3 mice were surgically extracted, rinsed in PBS and deposited in 12 wells microplates containing a specific medium composed of RPMI 1640 with 25 mM HEPES supplemented with 15% fetal bovine serum and 100 U/ml penicillin, 100 lg/ml streptomycin (GIBCO). Microplates were incubated at 37 C in 5% CO2/95% air with or without CamKII inhibitor (KN93). We checked that nerves anatomy was not affected by incubation (Supplementary Figure 1) as we already described (Bordignon et al., 2013). Lucifer yellow (1 mM) were added and nerves incubated during 4 h. Medium is removed, nerves rinsed with PBS and placed in 96 wells plates containing 200 μ l of medium. Fluorescence was recorded using a Perkin Elmer Victor 4 microplaque reader (excitation: 405 nM, emission: 535 nM).

### Pim1 inhibitors and biological evaluation

Two Pim1 inhibitors have been used. Pim-1 inhibitor 1 (p1) is a 3-Cyano-4-phenyl-6-(3-bromo-6-hydroxy)phenyl-2(1H)-pyridone,2-Hydroxy-3-cyano-4-phenyl-6-(3-bromo-6-hydroxyphenyl)pyridine (Calbiochem, Millipore) and Pim-1 inhibitor2 (p2) is a 4-[3-(4-Chlorophenyl)-2,1-benzisoxazol-5-yl]-2-pyrimidinamine (Tocris, biochemicals). Biological analysis are described in this paragraph.

### Animal treatment

Transgenic animals received intraperitoneal injections, either with a placebo (0.9% physiological serum) or with KN 93 (1.5 mg/kg). Treatment took place every day for 1 month.

### Rotarod test

Global evaluation of locomotor behaviuor was performed using the rotarod test, requiring motor coordination and balance control. A machine manufactured by Bioseb was used for this purpose. A previously used procedure (Mones et al., 2012) was adapted and two velocities (15 and 25 rpm) were used. Briefly, each mouse underwent the same 5-day procedure. The first 2 days were used to train the animals (five sessions of 2-min walking at a very low speed, i.e. five rotations per minute (r.p.m.)). The last 3 days were used to run the test sessions. Each day, the mice performed two series of five trials, a training speed with a 1-h rest period between the two series and a 5-min rest period between two consecutive trials. The software provided by Bioseb (Sedacom v1) was used to monitor the time the animals were on the rod.

### Statistical analysis

Statistical analysis was performed using Prism v5.0. Mann-Whitney and chi-square tests were used for trend analysis. The significance threshold was *p* < 0.05. ^*^p-value < 0.05, ^**^p-value < 0.01, and ^***^p-value < 0.001.

## Results

### Transgenic Gjb1 lines present genomic instability

The mutations G12S and S26L were introduced into the *GJB1* gene contained in a human BAC (RP11-485H3) using homologous recombination in *E. coli* (Warming et al., 2005). The recombinant BACs containing the mutated *GJB1* gene were injected into the oocytes of B6CBF1 mice as described previously (Huxley et al., 1996). Five transgenic founders were born; two contained the G12S mutation (G1, G2) and three the S26L mutation (S1, S2, S3).

In a preceding article, we explore these different lines (Mones et al., 2012). We observed polyploidy in mitotic spread and an increase in nuclear volume in all transgenic lines, a result of mitotic instability that is frequently caused by centrosome overduplication. In addition, immunostaining for γ-tubulin, a centrosome component, revealed an increase in the number of multi-centrosomal cells in 10–20% of transgenic fibroblasts whereas only 1–3% of the wild type cells presented with more than two centrosomes.

Two transgenic lines with the most copies, G2 (two copies) and S3 (three copies), and expressing the trangene, were selected for further experiments.

### Centrosomes overduplication is linked to CamKII activity

**Figure 1:**
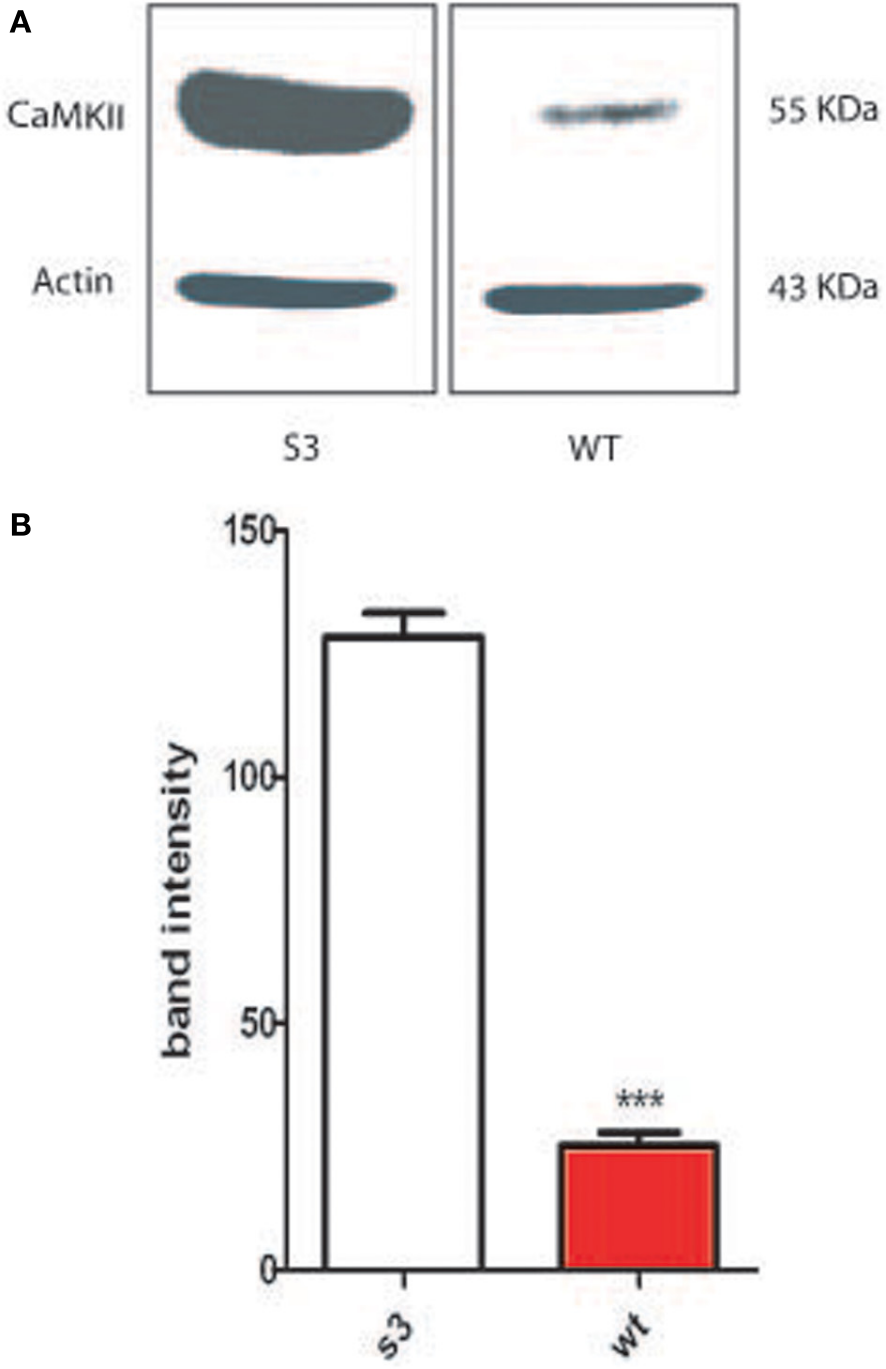
**Fibroblasts from wild type mice (WT) or transgenic lines (G2 or S3) were collected and cultured. (A)** Protein extract was obtained from WT and S3 fibroblasts, western blotted and probed with an antibody against phosphorylated CamKII. **(B)** Proteins from fibroblasts from either WT animals or S3 transgenic animals have been analyzed on an acrylamide gel and blotted. Membrane have been the probed with an antibody directed toward the phosphorylated form of CamKII. ^***^p-value < 0.001.

**Figure 2:**
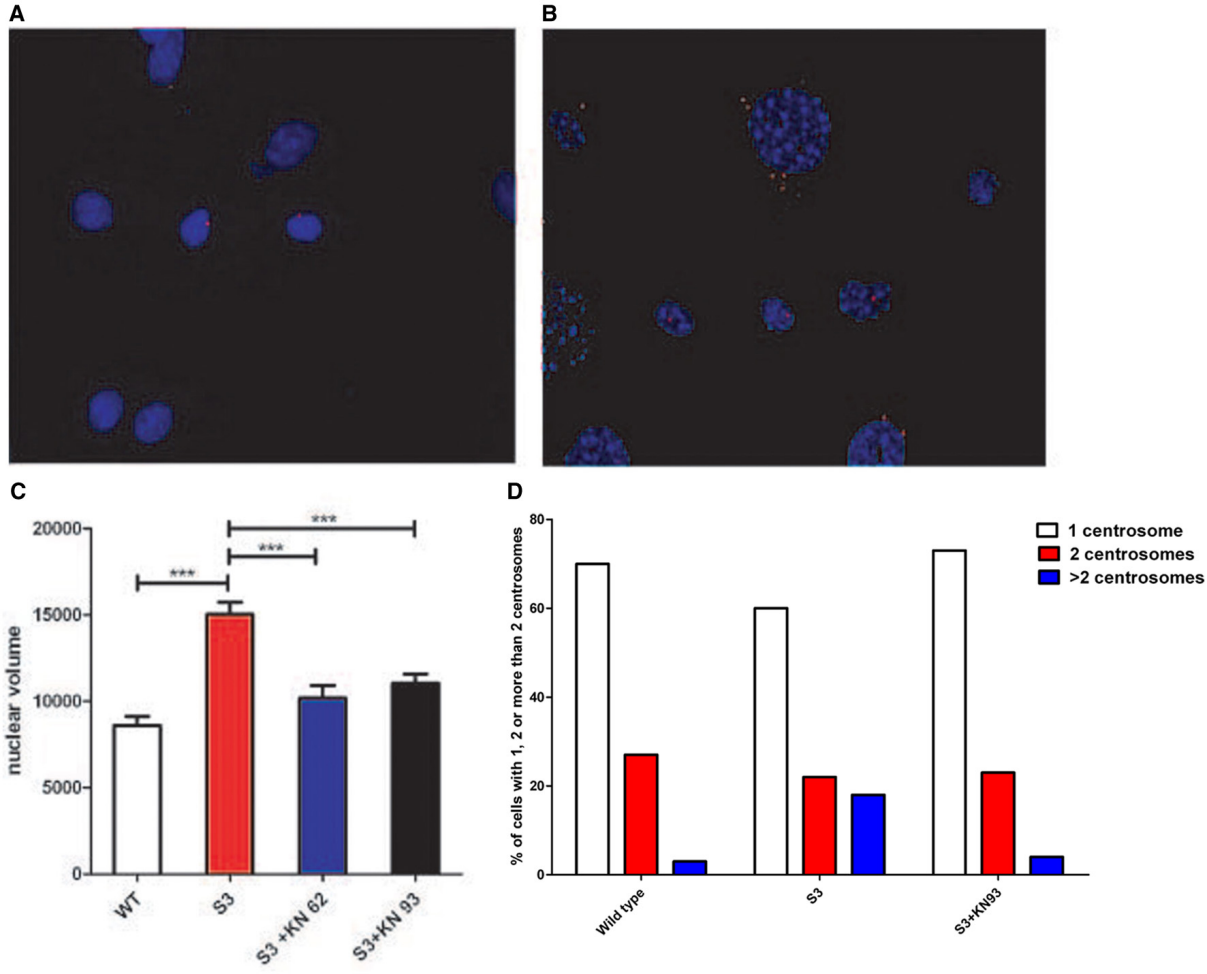
**(A)** Centrosomes of cultured WT fibroblasts were stained with an antibody raised against γ-tubulin (in red). **(B)** Centrosomes of cultured S3 transgenic fibroblasts were stained with an antibody raised against γ-tubulin (in red). An abnormal number of centrosomes could be observed. **(C)** Nuclear volumes of S3 fibroblasts and S3 fibroblasts treated either with KN93 or KN62 were evaluated after DAPI staining, using ImageJ software. **(D)** Fibroblasts from WT, S3, and S3 treated with KN93 were cultured and the percentages of cells with one centrosome (white), two centrosomes (red), and more than two (blue) were determined. This analysis has been repeated three times. ^***^p-value < 0.001.

**Figure 3:**
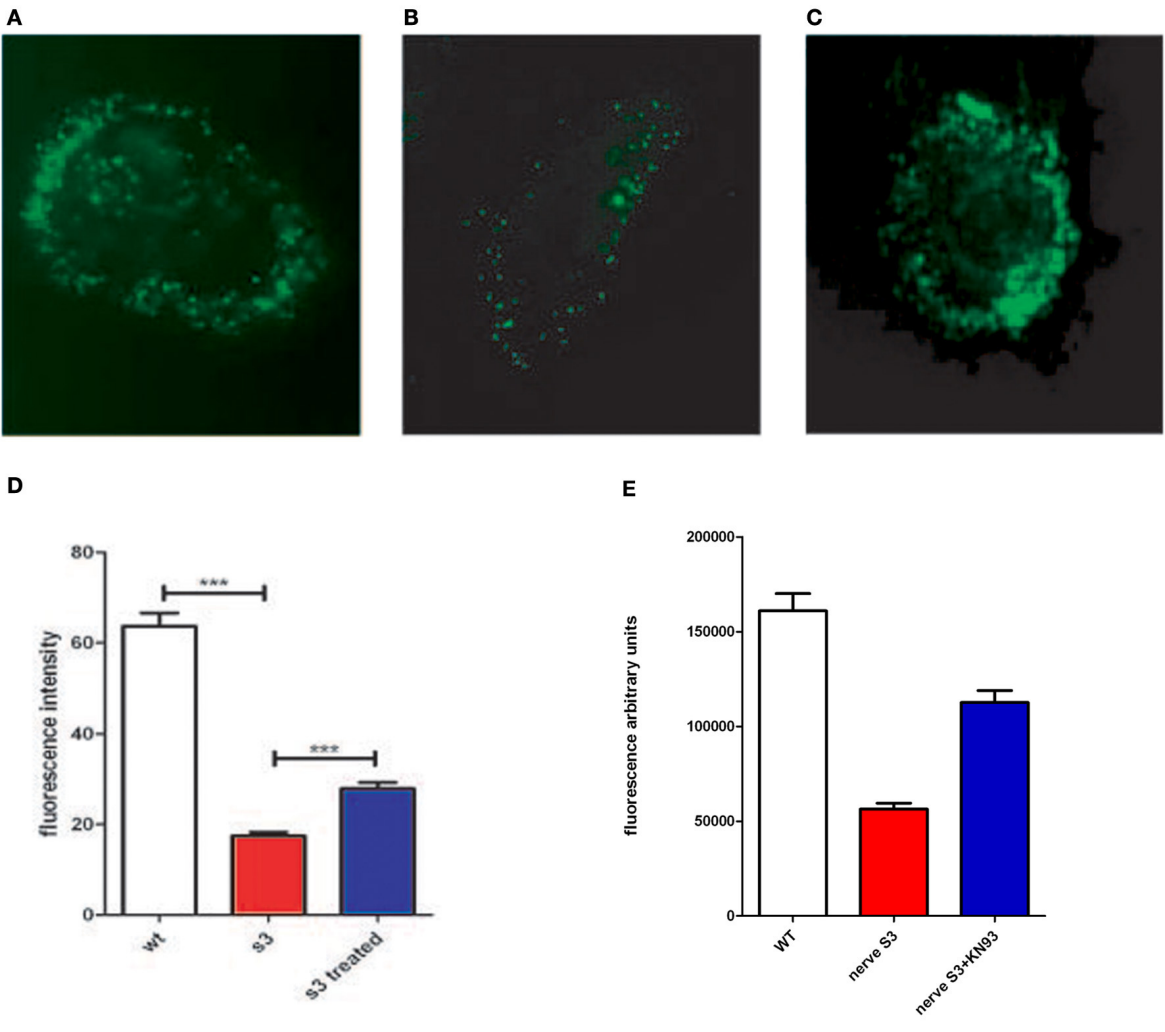
**Connexon activity of WT mouse fibroblasts (A), S3 transgenic mouse (B) and S3 incubated with KN93 (C), were incubated with LY for 2 h and examined under a fluorescent microscope. (D)** Connexon activity in fibroblasts from WT, S3, and S3 treated with KN93 was monitored in a 96 well plate assay, using lucifer yellow as a fluorescent dye (see methods). Fluorescence is in arbitrary units. **(E)** Sciatic nerves, isolated either from Wild type mice (WT) or from S3 transgenic mice, have been cultured for 24 h without or with KN93, a CamKII inhibitor. Connexon activity has been evaluated using internalization of LY (see Methods). ^***^p-value < 0.001.

In 2002, Matsumoto and Maller demonstrated that CamKII (Ca^2+^/calmodulin-dependent protein kinase II) activity and centrosome duplication are linked as CamKII overexpression led to centrosome over-duplication.As normal centrosome duplication is essential for genomic stability, we evaluated CamKII activity in cells from normal or transgenic lines. Myelinating mature Schwann cells are not proliferative, difficult to isolate and to culture, leading us to instead use fibroblasts isolated from transgenic lines. Figures 1A,B Show that the phosphorylated form of CamKII is strongly increased in S3 fibroblasts, indicating enzymatic overactivity in transgenic lines, and Figures 2A,B and 3B show the correlated centrosome overduplication. In order to validate these results and to investigate a method to rescue the cellular phenotype (centrosome overduplication), fibroblast cell lines derived from transgenic animals were incubated with two reference CamKII inhibitors, KN93 and KN62 (Tokumitsu et al., 1990; Sumi et al., 1991). In addition, KN92, a very close analog of KN93 differing only by an OH side chain but presenting no CamKII inhibitory activity, was used as a negative control. In the absence of CamKII inhibitor, all transgenic fibroblasts presented aneuploidy detected by evaluation of the nuclear volume. Figure 2C and Tables 1A,B show that treatment with either KN62 or KN93 resulted in restitution of normal nuclear volume. Similarly, evaluation of centrosome numbers in these lines show that treatment with either KN93 or KN62 revert them close to normal numbers (Figure 2D and Tables 2A,B). In contrast, abnormal centrosome number per cell is unchanged in cells treated with KN92. These results strongly suggest that involvement of Cx32 in chromosome stability is mediated through centrosome duplication, involving the CamKII signaling pathway.

**Table 1A:** **Nuclear volume correction**.

**Mouse lines**	***WT***	**S3**	**S3 + KN62**	**S3 + KN93**	**S3 + KN92**
Nuclear volume	8607 ± 508	15029 ± 690	10100 ± 729	11000 ± 540	15010 ± 550

*Fibroblasts from Wild type (WT) and S3 transgenic mouse have been cultured for 24 h with or without CamKII inhibitors (KN62 and KN93). KN 92 a molecule similar to KN93 but inactive on CamKII, has also been used. Nuclear volume has been evaluated using the Imagej software. Data have been analyzed using the Mann-Whitney test, demonstrating that differences are not statistically different between cells treated with KN92 and wild type but are highly statistically significant between non-treated and cells treated either with KN62 or KN 93. (p-Value < 0.0001). This experiment has been repeated three time*.

**Table 1B:** **Nuclear volume correction**.

**Mouse lines**	***WT***	**G2**	**G2 + KN62**	**G2 + KN93**	**G2 + KN92**
Nuclear volume	8607 ± 508	14000 ± 580	9950 ± 695	9900 ± 532	15000 ± 530

*Fibroblasts from Wild type (WT) and G2 transgenic mouse have been cultured for 24 h with or without CamKII inhibitors (KN62 and KN93). KN 92 a molecule similar to KN93 but inactive on CamKII, has also been used. Nuclear volume has been evaluated using the Imagej software. Data have been analyzed using the Mann-Whitney test, demonstrating that differences are not statistically different between cells treated with KN92 and wild type but are highly statistically significant between non-treated and cells treated either with KN62 or KN 93. (p-Value < 0.0001). This experiment has been repeated three time*.

**Table 2A:** **Number of centrosomes per cell**.

**Mouse lines**	***WT***	**S3**	**S3 + KN 62**	**S3 + KN 93**	**S3 + KN92**
1 Centro	79%	60%	62%	73%	60%
2 Centro	18	22	32	20	20
> Centro	3	18	6	7	20

*Fibroblasts from WT and S3 have been cultured with or without CamKII inhibitors. Centrosome number per cell has been evaluated as described in methods. Results have been analyzed using the Chi2 trends for tendency test demonstrating that data distribution is highly significant between treated and non-treated cells (p-value < 0.0001). This experiment has been repeated three time*.

**Table 2B:** **Number of centrosomes per cell**.

**Mouse lines**	***WT***	**G2**	**G2 + KN 62**	**G2 + KN 93**	**G2 + KN92**
1 Centro	79%	61%	70%	71%	60%
2 Centro	18	27	24	21	24
> Centro	3	12	6	8	16

*Fibroblasts from WT and G2 have been cultured with or without CamKII inhibitors. Centrosome number per cell has been evaluated as described in methods. Results have been analyzed using the Chi2 trends for tendency test demonstrating that data distribution is highly significant between treated and non-treated cells (p-value<0.0001). This experiment has been repeated three time*.

### Connexon activity is impaired in transgenic lines and corrected by CamKII inhibitors

To test the effect of CamKII inhibitors on connexon activity impaired by Cx32 mutations we analyzed connexon activity in WT and transgenic fibroblasts with KN93. The assay uses lucifer yellow dye (LY) which becomes fluorescent upon internalization by cells, thus specifically indicating connexon activity. If CamKII overstimulation is linked to anomalies of connexon activity, partial inhibition of CamKII would be expected to restore some, if not all, connexon activity. We observed that connexon activity is severely reduced in transgenic cells, and is significantly improved upon treatment with KN93 (Figures 3A–D, and Table 3). These data suggest that CamKII activity and connexon activity are probably linked.

**Table 3:** **Fibroblasts from *WT* and S3 (treated or not with KN93) have been cultured for 2 days in 96 wells microplaques**.

**Mouse lines**	***WT***	**S3**	**S3 + KN93**	**G2**	**G2 + KN93**
LY intensity	63 ± 17	17 ± 5	28 ± 9	24.5 ± 6	43 ± 7

*Lucifer yellow has been then added to the medium and fluorescence monitored after 2 h (cell auto fluorescence and fluorescence of LY without cells, have been evaluated and substracted). This experiment has been repeated three time*.

To test these results on *in vivo* phenotype, we developed a method to test connexon activity in sciatic nerves surgically removed from mice (for details see Methods). In brief, nerves were surgically removed, rinsed, and incubated in a specific medium for 24 h with or without CamKII inhibitor (KN93) and connexon activity evaluated using LY internalization. To account for variations in tissues compared to cells, testing was done on a larger number of nerves (12 nerves from 6 individuals). Results are presented in Figure 3E, and nerve integrity after incubation presented in supplementary Figure 1. We observed that connexon activity is reduced in S3 nerves and partially restored in S3 nerves treated with KN93. These data demonstrate that reduction, and partial restoration, is not restricted to fibroblasts but also observed in sciatic nerves.

### Is the kinase Pim-1 involved in mitotic instability of Gjb1 transgenic lines?

**Figure 4:**
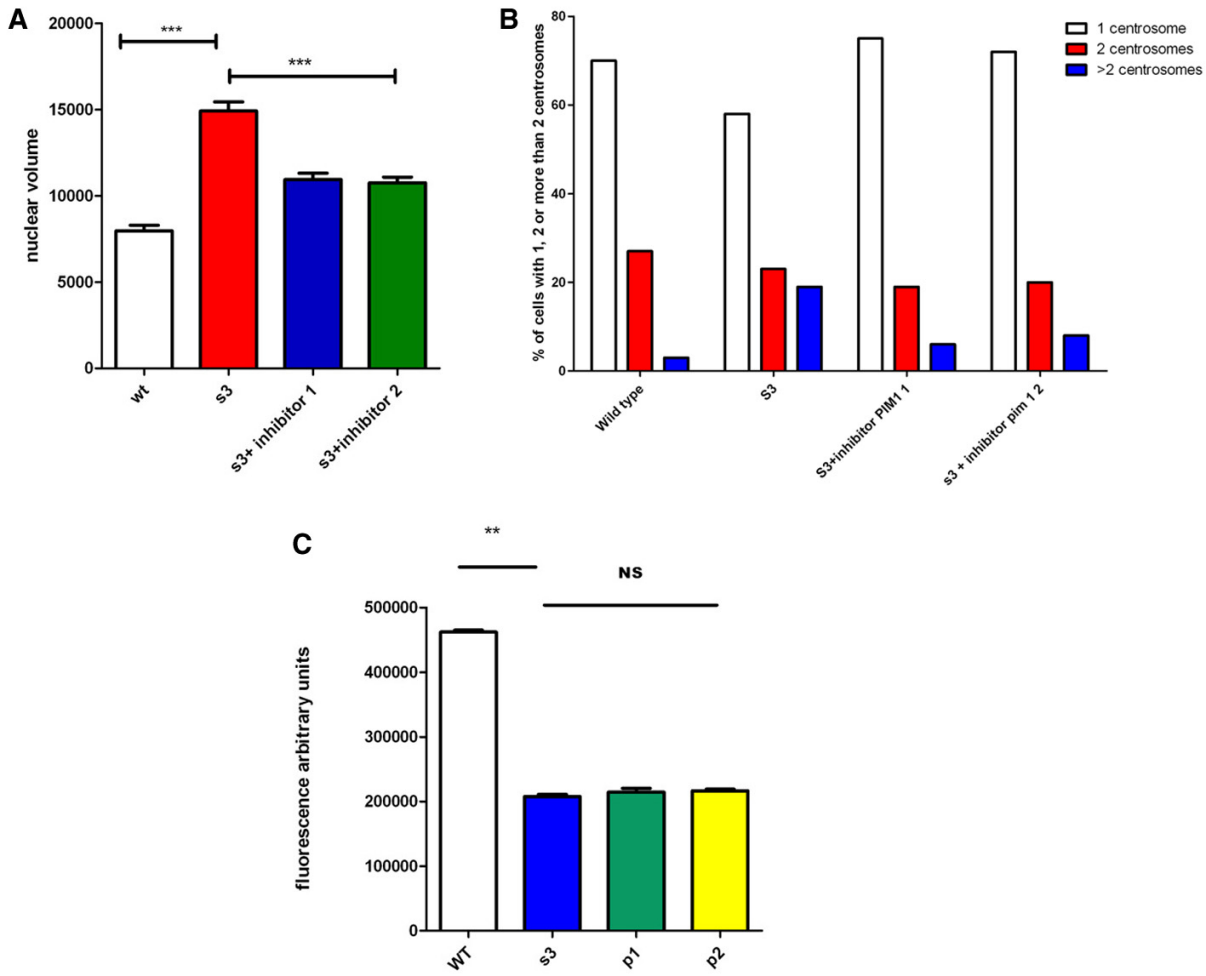
**Impact of Pim1 inhibitors on genomic instability and connexon activity of transgenic cells**. Cells from wild type animals (WT) or from S3 transgenic line, have been incubated without or with 2 different Pim-1 inhibitors (P1, and P2, see Methods). Nuclear volume **(A)**, cenrosome over duplication **(B)** or connexon activity **(C)** have been evaluated. ^**^p-value < 0.01; ^***^p-value < 0.001.

A recent report (Gao et al., 2013) indicated that CamKII inhibitors, directly or indirectly, inhibits several other kinases, including Pim1, a kinase that has been associated with genomic instability in cancers (Roh et al., 2003, 2005). We incubated cells from S3 transgenic lines with and without two different Pim1 inhibitors (see methods). Nuclear volume, centrosome over-duplication, and connexon activity were analyzed and the results are presented in Figure 4. We observed that incubation of cells with Pim1 inhibitors significantly corrects nuclear volume as well as centrosome overduplication. However, a comparable rescue of connexon activity was not observed.

### Degradation of *in vivo* behavioral phenotype is lowered by treatment with CamKII inhibitors

Charcot-Marie-Tooth type X is a clinically heterogeneous group, with great variability of phenotypes, mainly involving anomalies in the peripheral nervous system with both demyelinating and axonal features (Bergoffen et al., 1993; Hanemann et al., 2003) However, several families have been described where a mutation of Cx32 segregates and presents a central nervous phenotype (Hanemann et al., 2003; Stancanelli et al., 2012; Al Mateen et al., 2013).

**Figure 5:**
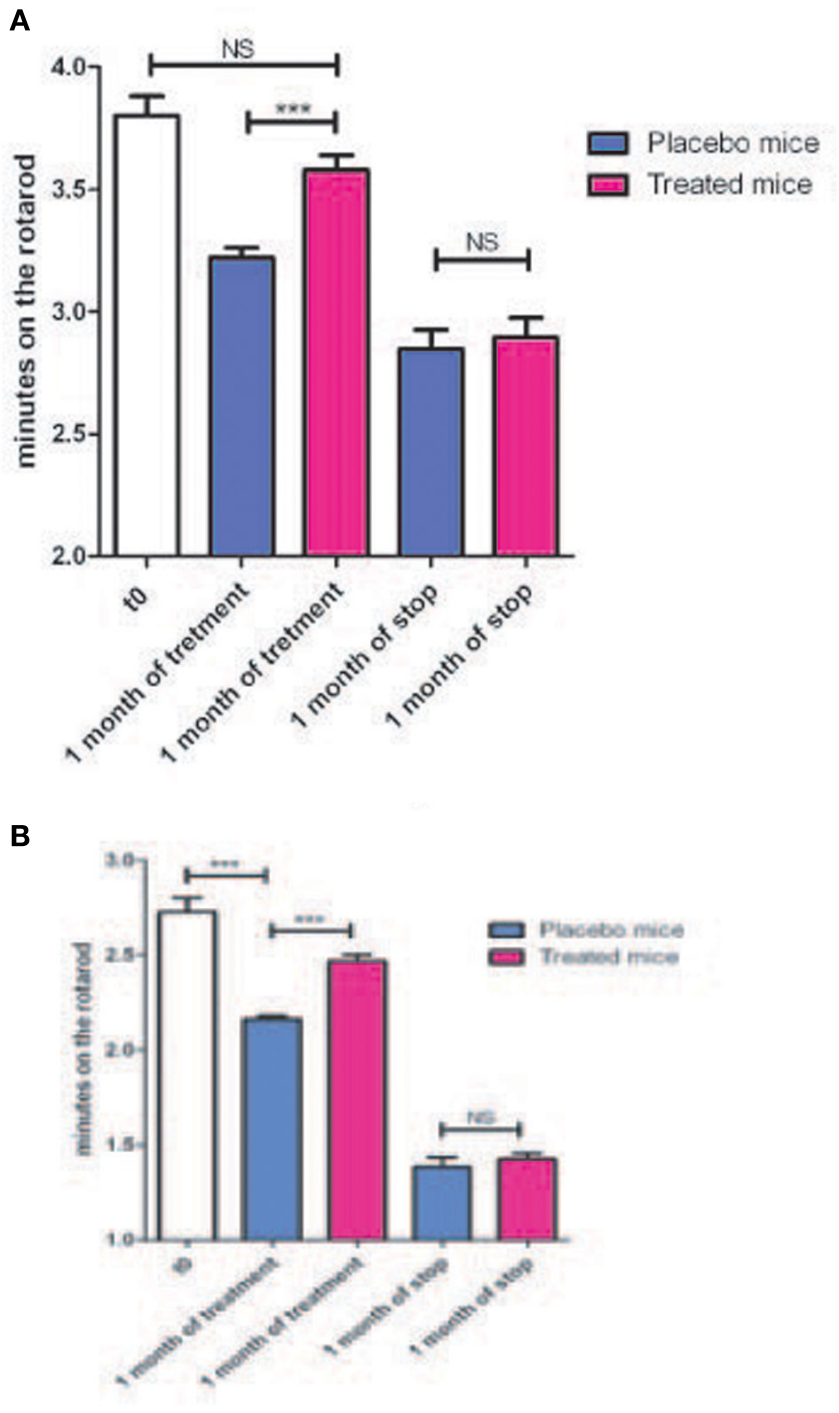
**(A)** Nine months old mice from the G2 line were treated either with a placebo (4 animals) or with soluble KN93 (4 animals, 1.5 mg/kg, i.p., 1 administration per day) for one month and performances on the rotarod evaluated. Treatment was then stopped and locomotor performances evaluated one month later. **(B)** Same as in **(A)** but using the S3 line. ^***^p-value < 0.001.

Therefore, we analyzed the impact of KN93 treatment on the behavioral phenotype already observed (Mones et al., 2012) in our transgenic models using the rotarod test. The S3 and G2 transgenic mouse lines present impairment appearing at about 7 months of age and progressing with age (Mones et al., 2012). These lines were treated with a soluble form of KN93 (1.5 mg/kg i.p. per day) and, as shown in Figures 5A,B and Tables 4, 5, resulted in significantly lowered progression of the phenotype. When KN93 treatment was stopped after 1 month, rapid degradation of the phenotype ensued. In contrast, placebo-treated animals still presented progressive degradation of the phenotype with age.

**Table 4:** **Mouse from the G2 transgenic line have been treated either with a placebo (4 animals) or with soluble KN93 (4 animals)**.

**G2 line**	**T0**	**placebo**	**treated**	**placebo + 1 month arrest**	**treated + 1 month arrest**
Mean minutes on rotarod	3.90	3.20	3.6	2.84	2.9
St error	0.2	0.15	0.22	0.28	0.28

*Locomotor performances of the animals have been evaluated using the rotarod test after one month of treatment. Performances of WT and G2 treated with KN93, are not statistically different. On the contrary performances of G2 animals treated either with a placebo or with KN93 are highly statistically significant (p-value < 0.0001). Treatment has been stopped for 1 month and rotarod performances evaluated. Rotarod performances of KN 93 treated or placebo treated degrade and are not statiscally different*.

**Table 5:** **Mouse (4) from the S3 transgenic line have been treated either with a placebo or with soluble KN93**.

**S3 line**	**T0**	**placebo**	**treated**	**placebo + 1 month arrest**	**treated + 1 month arrest**
Mean minutes on rotarod	2.7	2.1	2.5	1.5	1.41
St error	0.28	0.13	0.11	0.23	0.29

*Locomotor performances of the animals have been evaluated using the rotarod test after one month of treatment. Performances of WT and S3 treated with KN93, are not statistically different. On the contrary performances of S3 animals treated either with a placebo or with KN93 are highly statistically significant (p-value < 0.0001). Treatment has been stopped for one month and rotarod performances evaluated. Rotarod performances of KN 93 treated or placebo treated degrade and are not statistically different*.

## Discussion

We recently demonstrated (Mones et al., 2012) that connexin 32, involved in the X-linked form of Charcot-Marie-Tooth disease, is involved in mitotic stability. Expression of a mutated allele as wellas deletion of Gjb1 resulted in mitotic instability in our transgenic line, in cells from animals (Mones et al., 2012). The same situation is observed in CMTX patients, that present point mutations (frequent), duplication or deletion (rare) of Gjb1. We suggested here that this instability is due to CamKII overexpression, leading to centrosome overduplication. Matsumoto and Maller (2002) demonstrated the relation between calcium flux, CamKII activity, and centrosome duplication.

Moreover, Torok et al. (1997) identified two calmodulin-binding amino-acid sequences in connexin 32 and provided evidence that calmodulin may function as an intracellular ligand, regulating Ca2+ dependent intercellular communication across gap junctions. An examination of these specific calmodulin-binding sites identified two regions, one at the N-terminus and another one at the C-terminus, showing calcium-independent calmodulin-binding properties (Ahmad et al., 2001). Using a truncation mutant Cx32D215 they demonstrated that amino acid sequences in the third transmembrane domain and a calmodulin-binding domain in the cytoplasmic tail of Cx32 are likely candidates for regulating connexin oligomerization. Finally Dodd et al. demonstrated that this physical proximity of Cx32 and CamKII has a physiological role (Dodd et al., 2008).

It is therefore likely that pathological mutations in Cx32, associated to CMTX, result in mitotic instability through CamKII overexpression leading to centrosome overduplication. It has been observed by us (Mones et al., 2012) and by the European Mitocheck project (www.mitocheck.org) that a lowering of Cx32 expression or expression of a mutated isoform resulted in perturbation in cell division. Control of cell division is a key event in Schwann cell division. Moreover, perturbation of cell division has been associated to defects in myelination in experimental models as well as in patient nerves.

In this report, we demonstrated that treatment with CamKII inhibitors (KN62 or KN93) resulted in a partial rescue of the cellular phenotype (abnormal centrosome overduplication, mitotic instability, and connexon activity). This strongly suggests that CamKII activity, genomic instability and connexon activity are linked as defects in one of these processes led to defects in the others. In addition, rectifying one of these processes led to restoration of the others. This is consistent with references cited above, demonstrating physical proximity of connexins and CamKII in the cellular membrane. In addition, *in vivo* KN93 treatment of CMTX-related transgenic mice, although not fully correcting the degenerative behavioral phenotype, significantly lowered the degradation of performances. It has been suggested that it is through loss of function that pathogenicity of Cx32 mutations plays a role (Sargiannidou et al., 2009). However, CMTX phenotype expressed in human females suggests that a mutated allele expressed together with a normal allele (there is no bias in chromosome X inactivation regarding Gjb1) still leads to a pathological phenotype. This is consistent with our observations and model.

However, there is obviously a difference between the Cx32/CamKII complex and mitotic instability. Among the different kinase targets of KN inhibitors, we select the Pim1 serine/threonine kinase (Gao et al., 2013) because it is involved in mitotic instability in cancer cells (Roh et al., 2003, 2005). This instability is linked to anomalies in the mitotic spindle and centrosome overduplication (Roh et al., 2003). Treatment of our transgenic cells with inhibitors of Pim1 significantly corrects genomic instability, suggesting that genomic instability in cells expressing mutated alleles of Gjb1 could involve Pim1. However, since CamKII is a membrane protein but Pim1 has been located in cytoplasm and nucleus, this suggests a downstream role for Pim1. In addition, if anomalies in the Cx32/CamKII complex act on Pim1, a proto-oncogene, it could explain observations reporting high incidence of tumors in Cx32 null mice (Temme et al., 1997). However, we did not observe a significant correction of connexon activity with Pim1 inhibitors, suggesting that Pim1 is likely a downstream player.

*In vivo* KN93 treatment of transgenic mice, although not fully correcting the degenerative phenotype, significantly lowered the degradation of performances of transgenic mice observed in rotarod tests. We did not know how anomalies in CamKII, due to mutations in Cx32, could be involved in anomalies in myelination and maturation of glial cells. However, a recent paper (Waggener et al., 2013) could give a clue to answer the question. This group demonstrated that CamKIIβ regulates maturation of oligodendrocytes in CNS and myelination, using mice knockouts for this gene. This function is probably mediated through a cytoskeleton-stabilizing role. We infer that anomalies in mitotic spindle, derived from the cytoskeleton, are also involved in mitotic instability. We thus postulate that a cascade including Cx32-CamKII-Pim1 is involved in PNS maturation and myelination. Anomalies in this cascade lead to reduction in myelination and Schwann cells maturation.

Regarding therapeutic development, it is too early to apply directly our results to the human pathology. Additional work needs to be performed, specially using patients cells to validate the phenomenon in human pathology. Toward this end, our work reported here provides the first viable template to revert CMTX phenotype in humans.

### Conflict of interest statement

The authors declare that the research was conducted in the absence of any commercial or financial relationships that could be construed as a potential conflict of interest.
