# Supplementary material for: CamKII inhibitors reduce mitotic instability, connexon anomalies and progression of the in vivo behavioral phenotype in transgenic animals expressing a mutated Gjb1 gene
Source: Front Neurosci. 2014 Jun 13;8:151. doi: 10.3389/fnins.2014.00151 (PMC4056282; doi:10.3389/fnins.2014.00151)
Supplement: Supplementary file 1 [file Presentation1.PDF]

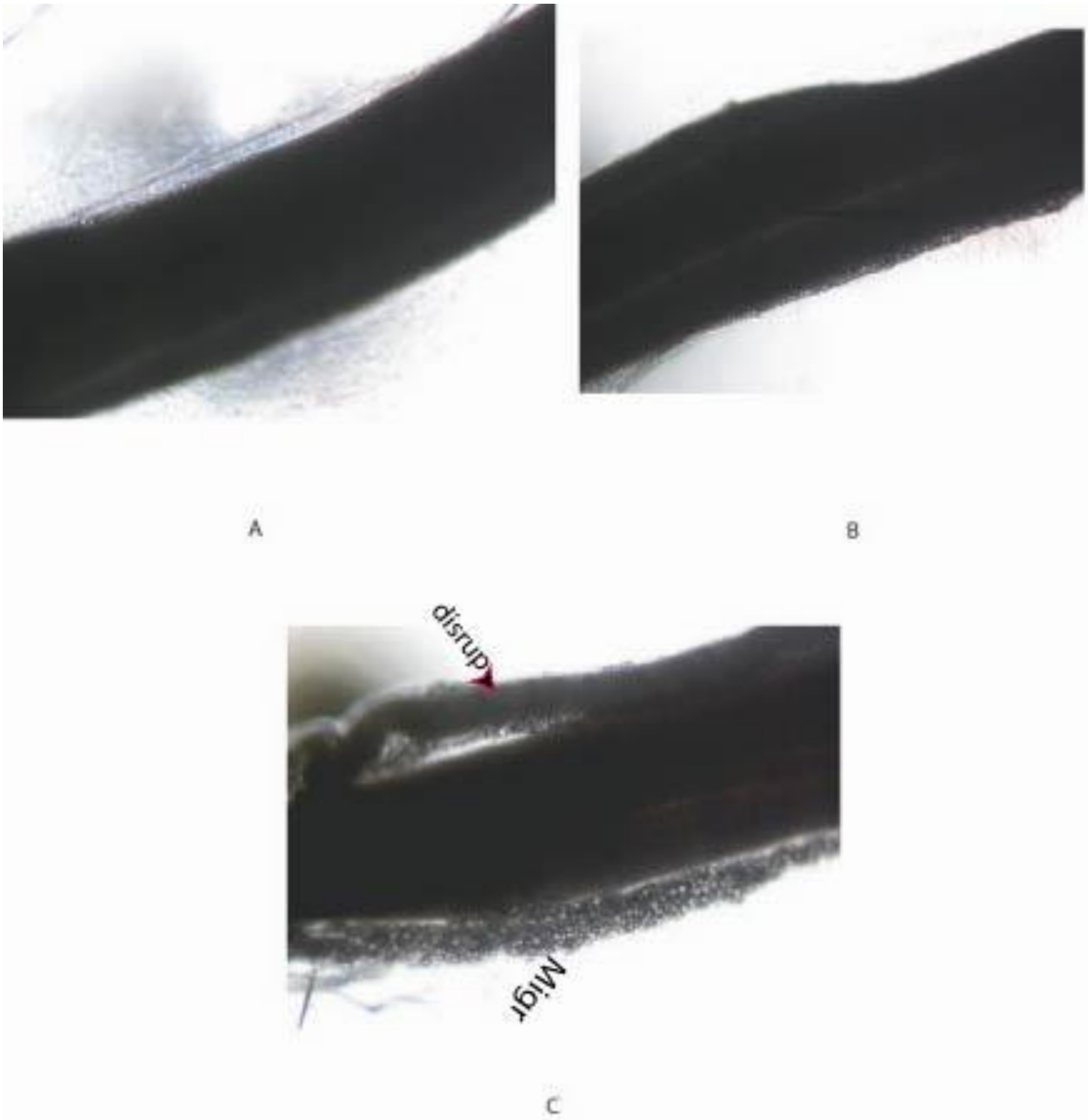

Supplementary figure1. Sciatic nerves from normal and transgenic mice, were sugically extracted and resuspended in a specific medium (see methods). Aspects of these nerves are presented in A. After 12 h, nerves have been incubated with LY (B). We present in C, the aspect of nerves incubated during 2 days in the medium. migraton of cells outside the nerve and partial disruption of structures could be observed, at the contrary of nerves presented in B (only 12h).
